# Supplementary material for: Tuning collective behaviour in zebrafish with genetic modification
Source: PLoS Comput Biol. 2024 Oct 28;20(10):e1012034. doi: 10.1371/journal.pcbi.1012034 (PMC11542821; doi:10.1371/journal.pcbi.1012034)
Supplement: S1 Text — The differences of the otoliths between the fish and the wildtype zebrafish, the analysis on the swimming location of the fish, the effect of parameter α in the simulation, more details on the shape–bending analysis of the zebrafish, and more details on the experimental procedure. (PDF) [file pcbi.1012034.s001.pdf]

SUPPLEMENTARY MATERIAL

Tuning Collective Behaviour in Zebrafish with Genetic Modification

Yushi Yang
Bristol Centre for Functional Nanomaterials, University of Bristol, Bristol, UK

Abdelwahab Kawafi, Qiao Tong, and Chrissy L. Hammond
Department of Physiology, Pharmacology, and Neuroscience, Medical Sciences, University of Bristol, Bristol, UK

Erika Kague
Department of Physiology, Pharmacology, and Neuroscience, Medical Sciences, University of Bristol, Bristol, UK and Institute of Genetics and Cancer, Centre for Genomic and Experimental Medicine, University of Edinburgh, Crewe Road South, Edinburgh, UK

C. Patrick Royall
Gulliver UMR CNRS 7083, ESPCI Paris, Université PSL, Paris, France.

I. BIOLOGICAL DIFFERENCE BETWEEN *COL11A2*-/- FISH AND WILD–TYPE FISH

In the main text we focused on the vertebral structure of the *col11a2*-/- mutant fish (Fig 1), since there is a direct link between the backbone and the bending of the fish body, as we hypothesized that the col11a2 -/- zebrafish to bear more pain when bending, because of their defective spines. Furthermore the lack of bending resulted in the change of their swimming posture, on the single–fish level, as well as the collective behaviour on the group level. Here we give some more details and also discuss additional biological difference between the mutants and the wildtype fish, in particular the structures of the otoliths, a small bone structure inside the inner ear of the zabrafish.

In addition to the IVDs, the *col11a2* gene also affects the otoliths of the zebrafish significantly. Such an effect on the small rod–like structure inside the inner ear of zebrafish (see Fig A for the location of the otoliths) is expected, because *col11a2* mutations in humans cause Stickler Syndrome, which is also associated with changes in hearing [1, 2]. Abnormalities in otolith formation can lead to swimming behaviour impairment in zebrafish, commonly associated with looping and rolling behaviour [3]. Although such behaviour was not observed in our experiments, the differences in the otolith due to the lack of functional *col11a2* could also contribute to the behavioural changes in our main text.

We analyzed the morphology of otoliths in adult *col11a2*-/- (sample size $n = 10$) using μCT, by segmenting these small structures in the fully scanned fish image, so that the voxels corresponding to different otoliths are labelled with unique values. The segmentation task was carried out with a convolutional neural network (CNN) with a 2D U-Net architecture [4]. Specifically, we used the resnet34 as the backbone of our U-Net [5], and we used the softmax function as activation function for the final layer to achieve multiclass classification. We trained our model with 14 manually segmented images, by minimising the Tversky loss function [6] with the help of an Adam optimizer [7]. The project and hyperparameter training are accessible at https://github.com/wahabk/ctfishpy.

The morphology of the otoliths are very different between the wildtype fish and the mutant fish, as shown in Fig A (b) and (c). The shapes of three different otoliths: lagenals, sacculars, and utriculars, are well–rounded and symmetrical in the wildtype fish, as shown in Fig A (b). Notably, the utricars of the mutant fish have an irregular shape, as shown in Fig A (c), where the lagenal on the right hand side (the top one in the plot) having a significantly small volume. Amongest the ten characterized, we identified 8/10 of mutant fish with lagenar-saccular otolith fusion, where the two structures are merged into a single one, while none of the wildtype fish exhibited such fusion. In addition to the morphology, we measured the relative density of the otoliths against the density of standard hydroxyapatites for calibration of the μCT. The density values are shown in Fig A (a), where the otholiths in *col11a2*-/- had significantly lower densities. In summary, there is significant difference in the otoliths between the wt fish and the mutant fish, which may contribute to the collective behavioural feature of the mutant fish.

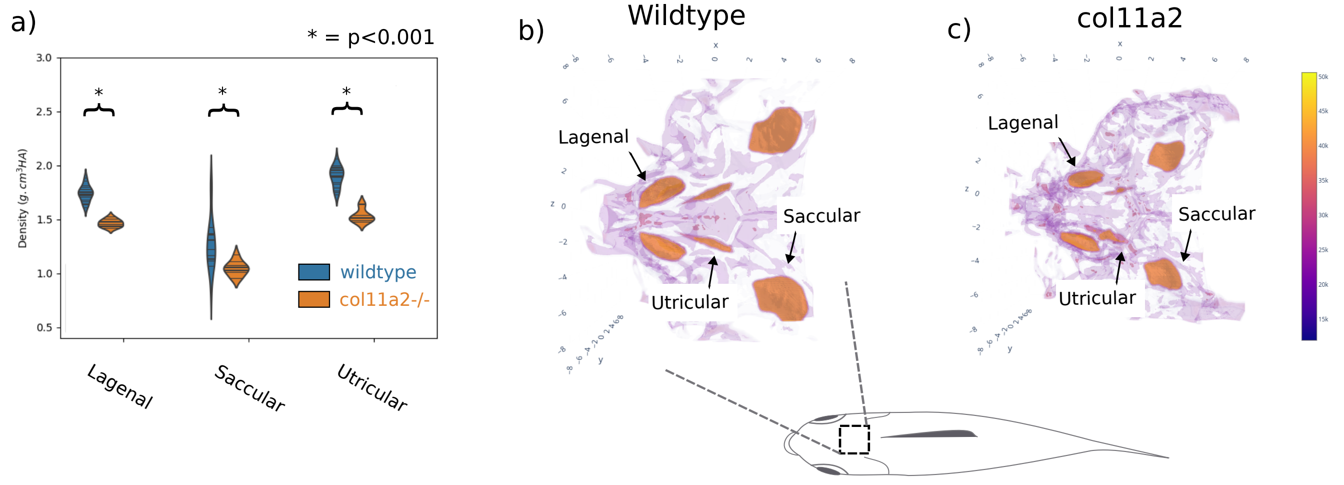

FIG. A. **Otolith analysis of wildtype and *col11a2*-/- zebrafish.** (a) Otolith density violin plots, density is quantified through calibrated μCT and u-net segmentations. N = 40, 23 for 6 and 12 month old wildtypes and N = 10 for *col11a2* -/- of both ages. Each horizontal line in the violin plots represents a data point. (b-c) Dorsal view volume renderings of 12 month old zebrafish otoliths, left to right (posterior to anterior): Lagenar, Utricular, and Saccular otoliths in order. Color represents 16 bit grayscale values thresholded below 12,000.

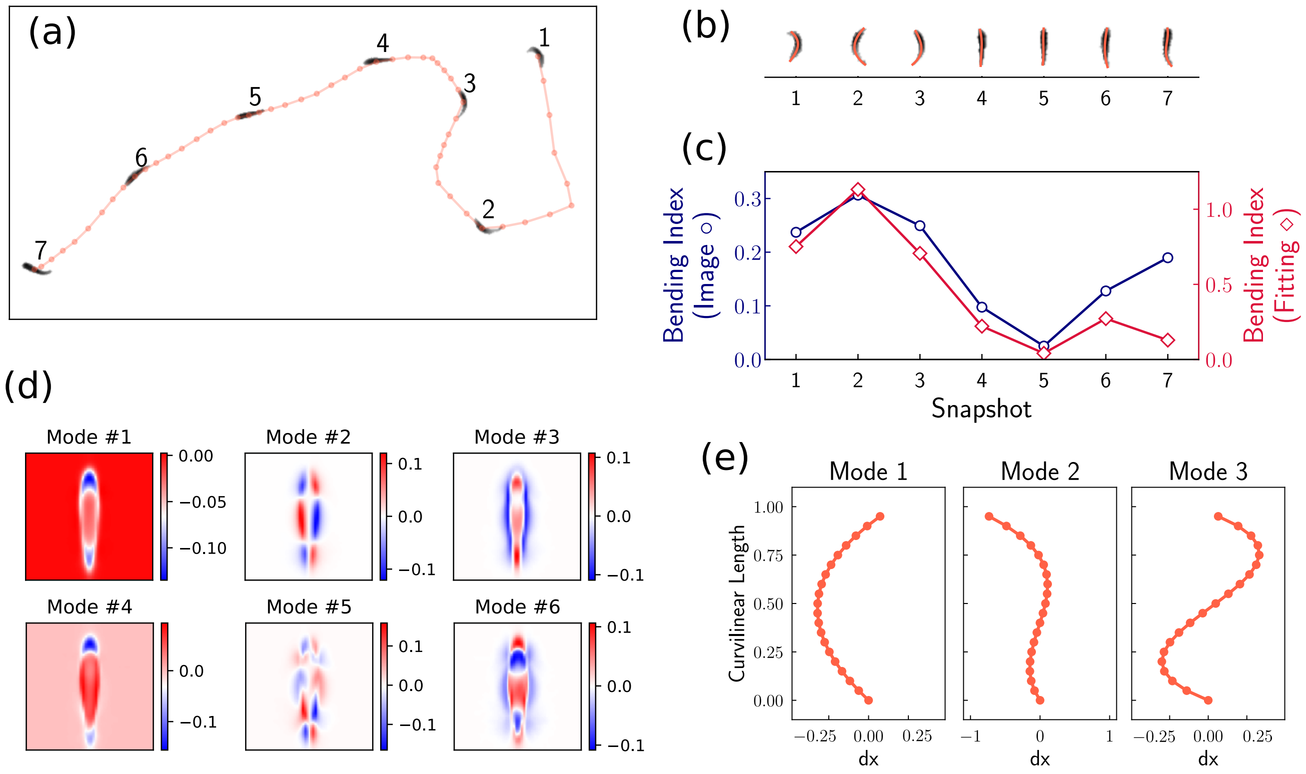

FIG. B. **The method for analysing the posture of the wt fish and *col11a2*-/-.** (a) The trajectory of a single wt fish in a quasi-2D environment in 3.6 seconds, plotted as a line with dots. Each dot represents a single frame captured every 1/15 s. Seven snapshots were selected, whose corresponding fish shapes are shown in the plot, featuring the different postures. (b) The *aligned* shapes highlighted in (a), represented as a 50×50 matrix. The alignment process removed the relative translation and rotation between different shapes, so that we could focus on the bending of the fish body. The solid line on top of the fish body represents a 3rd degree polynomial fit. (c) The bending index calculated from the shape images (○) and the fitted polynomial functions (◇). The higher the index, the more curved the fish body is. (d) The eigenvectors calculated from the PCA of the images, reshaped back to 50×50 arrays. (e) The first three eigenvectors calculated from the PCA of the fitting results.

II. DETAILED DESCRIPTION OF THE BENDING ANALYSIS

We carried out a detailed principle component analysis (PCA) on the fish shape in the quasi-2D environment. The typical fish shapes are illustrated in Fig B (a), where the shapes of a single fish in seven successive time points are plotted. To ensure that our result actually captures the morphological details, we analysed the data with two different methods. Firstly, we rotated these shapes so that they were aligned with the head pointing to the top, as illustrated in Fig B (b). These rotated shapes are represented by a 50×50 matrix, which are subjected to the PCA procedure in the main text. Briefly, we flatten the 50×50 matrix into a 1D array $\mathbf{x} \in \mathbb{R}^{2500}$, and we collected m shapes to get a matrix $\mathbf{X} \in \mathbb{R}^{m \times 2500}$. We then calculated the eigenvectors of the covariance matrix $\mathbf{C} = \mathbf{X}^{\top}\mathbf{X}$, and sorted these vectors according to their corresponding eigenvalues. The eigenvectors revealed the movement modes of the fish, when reshaped from a 2500 dimensional vector to a 50×50 matrix, as shown in Fig B (d). The first mode illustrated the differences in the body of fish individuals, and the second mode revealed the bending of the fish. (The images of the fish accquire a higher value from the projection of the image to the eigenvector when their body overlaps with the red region in the second mode, and a lower value if the fish bends to another direction.) The other modes revealed more morphological details of the fish, and notably the 5th mode shows the wiggling of the fish tail.

In order to study the bending, we projected the captured fish shape (Fig B) onto the second mode, and we took the absolute value of the projection as the bending index values, shown in Fig B (c) (blue data), where we named this method “image” because we performed PCA directly on the obtained images. The result in Fig B (c) is promising, as the images in which the fish appear more bent in Fig B (b) have a larger corresponding bending index values.

We also investigated other ways to analyse images to obtain data about the bending of the fish. Here, we fitted the rotated shapes in Fig B (b) with a third order polynomial function. We then interpolated the fitting curve, so that each curve was represented by 20 values, representing the deviation from the x axis (see Fig B (e) for examples). Having collected m fitting results, we obtained a matrix $\mathbf{X} \in \mathbb{R}^{m \times 20}$ which can be analysed with the PCA procedure, where we calculated the eigenvectors of the covariance matrix $\mathbf{C} = \mathbf{X}^{\top}\mathbf{X}$ to extract the principle components. These components reveal the major movement modes of the fish while they swim, and the first 3 modes are shown in Fig B (e). The first mode indicate the bending of the fish, while the remaining modes depict more detailed movement like the wiggling of the tail (mode 2) and the synchronised bending of the head and the tail (mode 3). We projected the fitted curve on the first mode, and obtained the bending index values, as shown in Fig B (c) (red data), where we termed this method “fitting” because we performed PCA on the fitted curve instead of the images. These “fitted” bending index values are consistent with the results obtained from the PCA on the images, and such consistency validated both methods. In the main text, we reported the results obtained with the image method because it involves less data processing and being easier to implement. In any case, the two methods yields very similar results, as shown in Fig C.

III. THE EFFECT OF THE α PARAMETER IN THE SIMULATION

In our modified Vicsek model, the parameter α controls the orientational inertia of the agents (Eq. 2). The agents with a higher α value would swim in a more ballistic fashion and therefore have a longer persistence length l_p. Collectively, a group of simulated agents have a lager value for the reduced persistence length, as shown in Fig D. It is obvious that the original Vicsek model, whose $\alpha = 0$, can not fit the experimental observation. The value of 0.63 yields the best fit with the experimental result, which is also exactly the same value that fits the experimental result of 50 wildtype zebrafish in our previous study [8].

IV. THE EFFECTS OF SWIMMING LOCATION

We observe a consistent difference in the spatial preference between the col11a2 mutant fish and the wild–type. The wild–type fish is more likely to swim near the bottom of the tank, where the ballistic swimming movement will be interrupted by the boundary. Therefore, it is possible that the wild–type fish exhibiting a lower polarization value simply because their otherwise ordered movements are prevented by the container. Such possibility is excluded by the results in Fig E, where we performed the calculation of orientation auto–correlation function (ACF) and the polarization while conditioning the location of the fish group. When the fish swims very close to the bottom of the tank, the ACFs for the wild–type and the mutant fish decay in a similar fashion, as shown in Fig E (a). However in all other conditions, we see a consistent behavioural difference between the col11a2 group and the wild–type group. The mutant fish takes longer time to change their moving directions (characterized by a slower decay of the ACF) and these mutant fish exhibited a more ordered movement (characterized by a larger polarization value).

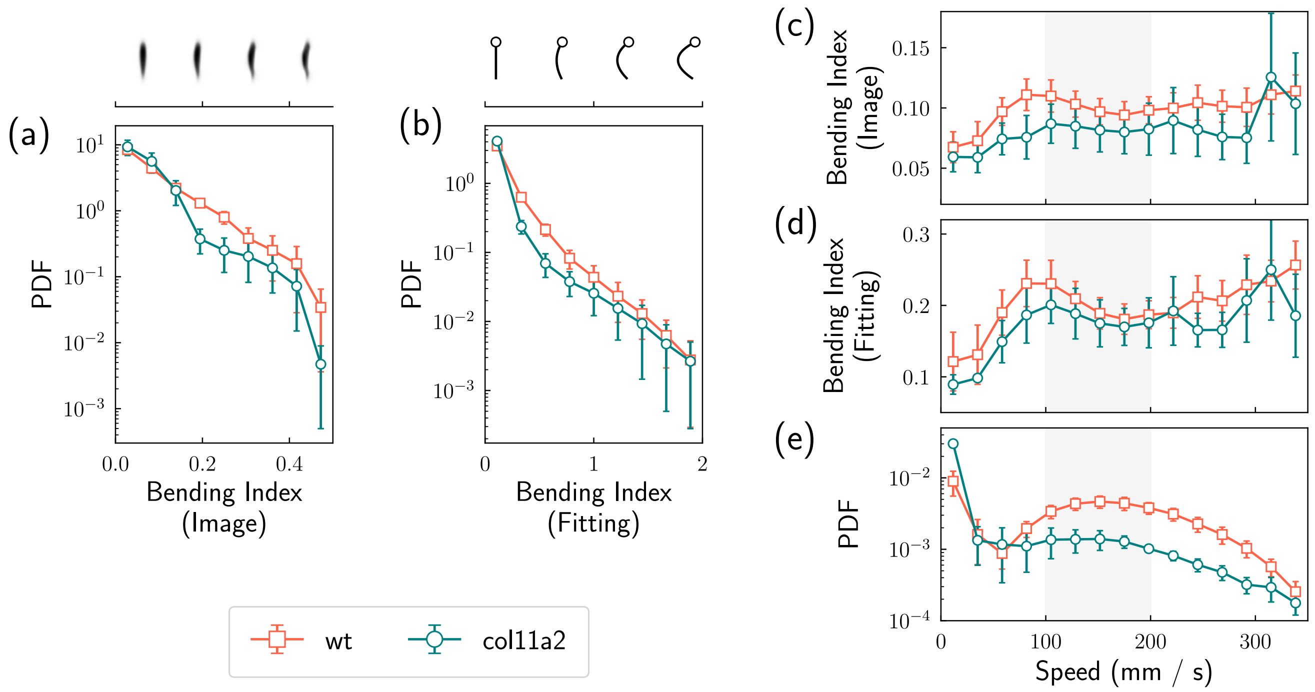

FIG. C. **The analysis of fish bending with two methods.** (a) The distribution of the bending index values calculated by performing PCA on the images of individual fish shapes. (b) The distribution of the bending index values calculated by performing PCA on the fitting result of the fish shapes . (c) The average bending indices, calculated from the images of the fish shapes, for the wt fish and the mutant fish at different speed values. (d) The average bending indices, calculated from the fitting result of the fish shapes, for the wt fish and the mutant fish at different speed values. (e) The distribution of the speed values for the fish.

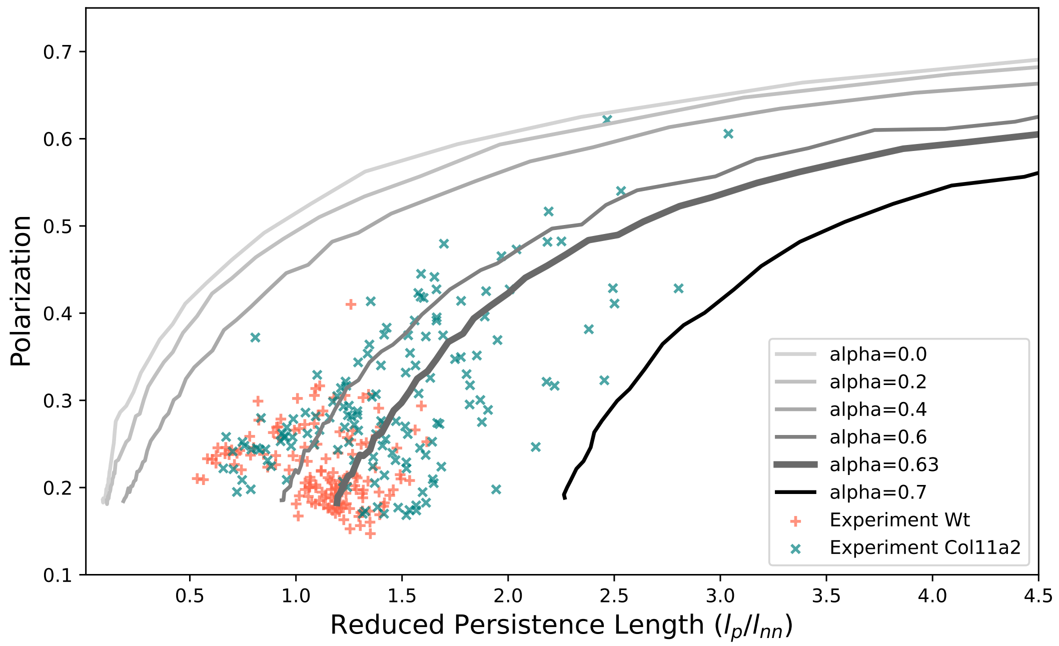

FIG. D. **The effect the α parameter in the modified Vicsek Model.** The solid lines are simulation results the modified Vicsek model (N=25) with different α values. The value of 0.63 yields the best match with the experimental observations.

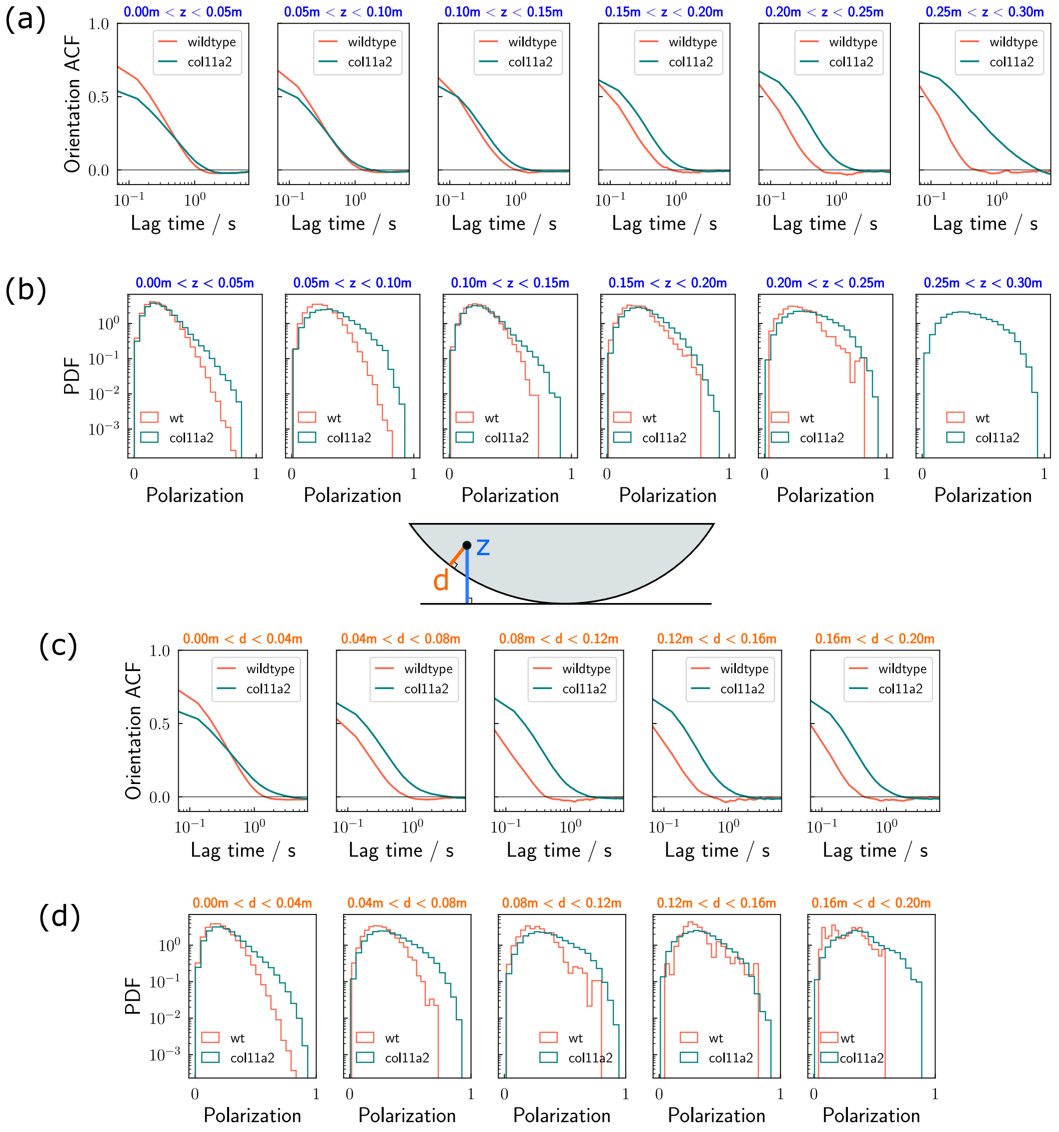

FIG. E. **The comparison between a group ($n = 25$) of wildtype zebrafish and col11a2 mutant zebrafish conditioned at different depths and distances to the tank.** (a) the averaged auto–correlation functions (ACF) of the orientation of the each individual fish in a group of twenty five. We discarded the orientation values whose depth values are not inside the targeted range, when calculating the ACF. (b) the distribution of polarization of 25 zebrafish, when the depth of the group center falls into different depth ranges. (c) the averaged ACF of the orientation of the each individual fish in a group of twenty five. We discarded the orientation values whose distances to the tank are not inside the targeted range, when calculating the ACF. (d) the distribution of polarization of 25 zebrafish, when the distance between the group center and the tank falls into different depth ranges. The insert in the center of the figure illustrates the geometrical definitions of “depth” and “distance to the tank”.

Notice that the conditioning for the ACF and the polarization are applied in two different manners. For the calculation of ACFs we masked out all the dot products of the orientation vectors, if their corresponding positions do not fall within the conditioned region. For the calculation of the polarization values, we discarded the frames where the group centers of the fish are not located within the conditioned region. One *should not* reach the conclusion that the mutant fish exhibits a higher polarization while having the same re–orientation time compared to the wild–type from the first column of Fig E (a) and (b). The ACF results and the polarization distributions are calculated from different data points because of their different conditioning methods.

TABLE S1. The experimental details of the 3D observations as well as the average orientational relaxation time measured in each experiment. For the first batch, the DOF of the fish is 2021-01-20. For the second batch, the DOF of the fish is 2021-05-13. In the one fish experiments, we observed the swimming behaviour of N different fish on the same date, and we note them as 1 $\times N$ in the "fish number" column.

Sample		Experiment Date	Fish Number	Relaxation Time (s)
Batch 1	wt	2021-03-03	25	0.2
	col11a2	2021-03-04	25	0.2
	wt	2021-03-17	25	0.27
	col11a2	2021-03-18	25	0.47
	wt	2021-03-25	1×12	0.18
	col11a2	2021-03-23	1×9	0.36
	wt	2021-03-30	25	0.20
	col11a2	2021-03-30	25	0.40
	wt	2021-04-13	25	0.20
	col11a2	2021-04-12	25	0.33
	wt	2021-05-12	25	0.13
	col11a2	2021-05-10	25	0.27
Batch 2	wt	2021-07-05	1×6	0.40
	col11a2	2021-07-05	1×6	1.08
	wt	2021-11-05	1×10	0.30
	col11a2	2021-11-23	1×10	0.55

V. DETAILS ON THE EXPERIMENT SCHEDULE

We repeated our observations several times to ensure that our conclusion on the effects of *col11a2* gene being repeatable. Specifically, we independently bred two batches of fish for our observation. In each batch, we crossed both wildtype zebrafish as well as *col11a2*-/- mutant fish on the same date, so that we can compare the different groups when they have have the same age. All of our 3D observation experimental details are summarised in Table S1.

For the 25 fish experiments, all the observations are performed on the fisrst batch, where the fish were bred on the 20th of January 2021. The fish group mature from 43 days post fertilisation (dpf) to 111 dpf as we carried out our observations, and the age–dependent change are illustrated in Fig F. In all different ages, the mutant fish expressed higher polarization values as well as a slower decay in the orientational auto–correlation function. The distribution of speed for the fish changed gradually from a bimodal to having a single peak for both mutant and wt fish, as the age of the fish increases. However, the relative difference between wt fish and the mutant fish is unchanged, where the wt fish exhibited longer tail in the speed distribution. Overall the relative difference between the wt fish and the *col11a2* mutant fish are consistent in all of our observations.

For the one fish experiments, we performed our observations in two different batches. And we observed 6 to 10 individuals in each experiment on the same date. The average orientational relaxation time for each experiment is written in Table S1, where the mutant fish consistently exhibited a larger relaxation time compared to the wt fish. The consistent difference among the wt fish and the *col11a2*-/- in different repeats and batches makes us confident about our finding that the phenotype (the fish take a longer time to change direction) is linked to the underlying genetic modification.

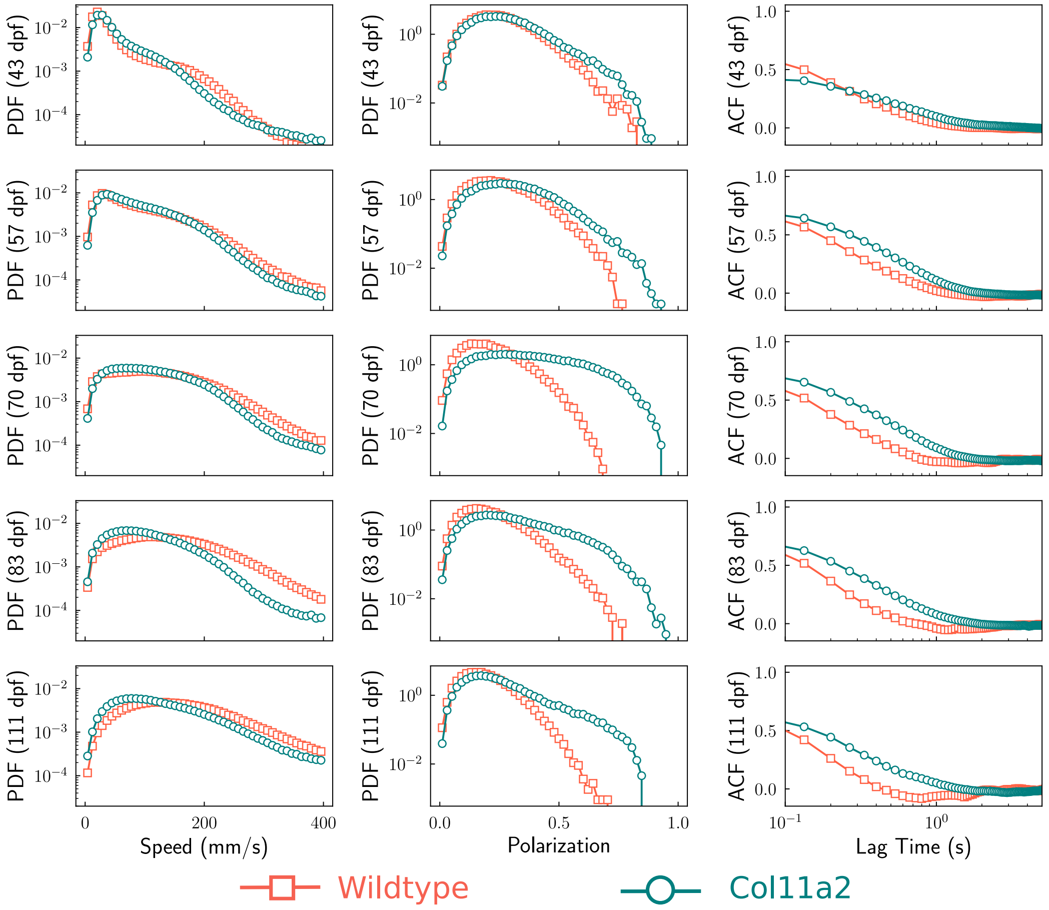

FIG. F. **The collective behaviour of 25 fish at different ages.** The different rows indicates the analysis resluts for the fish at different ages, from 43 day post fertilisation (dpf) to 111 dpf. The first column shows the distribution of the speed values for the wt and mutant fish. The second column shows the distribution of the polarization order parameters wt and mutant fish. The third column shows the auto–correlation function (ACF) of the orientations of the fish, for both wt and mutant groups.

[1] T. T. Whitfield, B. B. Riley, M. Y. Chiang, and B. Phillips, Developmental Dynamics **223**, 427 (2002).
[2] F. R. Acke, I. J. Dhooge, F. Malfait, and E. M. D. Leenheer, Orphanet journal of rare diseases **7**, 10.1186/1750-1172-7-84 (2012).
[3] I. A. Favre-Bulle, A. B. Stilgoe, H. Rubinsztein-Dunlop, and E. K. Scott, Nature Communications **8**, 630 (2017).
[4] O. Ronneberger, P. Fischer, and T. Brox, Lecture Notes in Computer Science **9351**, 234 (2015).
[5] K. He, X. Zhang, S. Ren, and J. Sun, Deep Residual Learning for Image Recognition (2015).
[6] S. S. M. Salehi, D. Erdogmus, and A. Gholipour, Lecture Notes in Computer Science **10541 LNCS**, 379 (2017).
[7] D. P. Kingma and J. Ba, Adam: A method for stochastic optimization (2014).
[8] Y. Yang, F. Turci, E. Kague, C. L. Hammond, J. Russo, and C. P. Royall, PLOS Computational Biology **18**, 1 (2022).
